# Assessment of the Safety and Efficiency of a Preperitoneal Continuous Infusion Using Bupivacaine after Abdominal Laparotomy in Digestive Carcinology

**DOI:** 10.1155/2023/8842393

**Published:** 2023-10-10

**Authors:** Hayat Ben-Saghroune, Mohammed Abdessadek, Sanae Achour, Youssef Kfal, Abderrahim El Bouazzaoui, Nabil Kanjaa, Hicham Sbai

**Affiliations:** ^1^Laboratory of Anesthesia-Intensive Care and Emergency Medicine, Medical Center for Biomedical and Translational Research, Faculty of Medicine and Pharmacy, University Sidi Mohamed Ben Abdellah, Fez, Morocco; ^2^Laayoune Higher School of Technology, Ibn Zohr University, Agadir, Morocco; ^3^Faculty of Medicine and Pharmacy, Biomedical and Translational Research Laboratory University Sidi Mohammed Ben Abdellah, Pharmaco-Toxicology Department, Hassan II University Hospital, Fez, Morocco; ^4^Central Medical Analysis Laboratory, Hassan II University Hospital, Fez, Morocco; ^5^Anesthesiology and Intensive Care Department A4, University Hospital Hassan II, Fez, Morocco; ^6^Anaesthesia and Critical Care Department, University Hospital of Tangier, Simulation Center Faculty of Medicine, University Abdelmalek Essadi, Tangier, Morocco

## Abstract

The purpose of this paper is to evaluate the safety and efficacy of continuous preperitoneal wound infiltration using bupivacaine after abdominal laparotomy in relation to plasma bupivacaine concentration and visual analog scale. Our study was performed on 60 adult patients with digestive cancer, operated at laparotomy, and randomized into two groups: bupivacaine and saline groups. The wound infiltration was through a multiperforated catheter along the scar. For the bupivacaine group, 0.25% bupivacaine was used; however, for the saline group, only saline (0.9%) was infiltrated. The pain was assessed by using the visual analog scale (VAS) in both groups. Plasma bupivacaine concentration was measured by high-performance liquid chromatography. The bupivacaine group had significantly lower postoperative morphine consumption and lower postoperative pain than the saline group (*P* < 0.0001). The majority of patients in the bupivacaine group had significant relief with the VAS scores of less than 3/10 cm at rest and 6/10 cm on mobilization. However, for the saline group, the VAS scores were higher than 6/10 cm either at rest or with mobilization. There was no clinical sign of toxicity and no technical complications for the bupivacaine group. Only eleven patients required morphine in this group, but the majority of patients received morphine at different doses in the saline group. Plasma bupivacaine was at very low concentrations. Overall, the current study has confirmed that continuous preperitoneal wound infiltration as postoperative analgesia is a simple, effective, and safe technique. It allows decreasing of morphine consumption and subsequently canceling their side effects.

## 1. Introduction

Recent evidence suggests that postoperative pain is the most common form of pain [1, 2]; this clinical manifestation constitutes a challenge that clinicians have been trying to solve for more than a decade, especially in major surgeries such as oncologic surgery, which causes severe acute and chronic postoperative pain. Postoperative pain management is a patient right and an absolute requirement to reduce the stress response after surgery [3]. For this reason, there are many analgesic procedures that can be used to treat postoperative pain such as systemic parenteral analgesia, central, and peripheral techniques. One of the peripheral analgesic techniques is wound infiltration, which plays a major role in postoperative pain prevention. Hence, the wound infiltration eliminates the parietal component of postoperative pain, which is the most common after abdominal surgery [4]. Furthermore, this analgesic technique is part of the concept of multimodal analgesia; it was developed to improve postoperative analgesia, reduce opioid consumption that may delay rehabilitation, and may also promote tumor proliferation [5].

In recent years, wound infiltration analgesia has become an important component of multimodal analgesia. For this purpose, the combination of various postoperative analgesic techniques uses the advantages of each method and minimizes side effects [6]. In the pain management techniques, the analgesia using wound infiltration requires the injection of local anesthetics and then the monitoring of the concentration of these drugs in the biological matrix. Despite its long clinical success, wound infiltration is associated with a number of side effects [7]. According to previous research data, the local anesthetics most commonly used in wound infiltration are amide-type local anesthetics, including ropivacaine, lidocaine, mepivacaine, and bupivacaine. Ropivacaine, as a widely used amide-type local anesthetic, has comparable efficacy to bupivacaine but lower systemic toxicity and shorter half-life to reduce the risk of plasma accumulation [8]. In clinical outcomes, levobupivacaine and ropivacaine have a pharmacological profile almost similar to that of bupivacaine, and the minimal differences reported between the three anesthetics are mainly related to the anesthetic potency, which is slightly different [9]. Bupivacaine is commonly used in wound infiltration in the management of postoperative pain after operations such as tumor resection abdominal surgery, orthopaedic surgery, and total gastrectomy.

The major risk involved with wound infiltration is the potential for drug toxicity. So far, there are few data on the value of continuous infusions of local anesthetics into the preperitoneal space after abdominal laparotomy. Given the lack of information on the bioavailability of local anesthetics, especially bupivacaine, administered preperitoneally and the risks associated with their systemic resorption, it seems important to have serum dosages during prolonged continuous infusions. Despite various articles reviewing the effects of wound infiltration technique on pain control and outcomes, only a few report complications and limitations [10–13]; systemic toxic effects and plasma concentrations of local anesthetics have not been studied in most cases.

Overall, the purpose of this study was to assess the safety and effectiveness of a preperitoneal continuous infusion using bupivacaine after abdominal laparotomy in association of plasma bupivacaine concentration and visual analog scale.

## 2. Methods

### 2.1. Evaluated Patients

The data of this prospective, observational, randomized, and control group study were based on the analysis of data from patients who received postoperative analgesia by wound infiltration of bupivacaine. These patients were suffering from digestive cancer and were operated for abdominal laparotomy in the central block of the Hassan II University Hospital, Fez, Morocco.

The study concerned all adult patients who were scheduled for an abdominal laparotomy because of digestive cancer, whatever its nature, and benefited from a continuous preperitoneal wound infiltration with 0.25% bupivacaine associated with other analgesics as part of the multimodal analgesic strategy.

In this work, the exclusion criteria were patients operated by laparoscopy, exploratory laparotomy, patients who received epidural analgesia as a per operative analgesia technique, and patients who did not receive this analgesic technique for the following reasons: severe hepatic and/or renal insufficiency, allergy to local anesthetics, or surgical site infection.

The primary outcome was the study of the effectiveness and safety of the analgesia technique by preperitoneal infiltration of bupivacaine in the wound. The secondary criteria were length of hospital stay, patient comfort, and patient satisfaction. After obtaining the approval of the Ethics Committee of the University Hospital of Fez (no. 17/19), all the patients included had given their written consent after oral information and handing over the explanatory document. The study was conducted on 60 cancer patients who were operated on by abdominal laparotomy and were divided into two groups: bupivacaine group and saline group.

Statistical analysis results are expressed as the mean ± SD. Normally distributed interval data are reported as mean and SD. Non-normally distributed interval and ordinal data are reported as median or range. Statistical tests used for the intergroup comparison were the *t*-test for quantitative data and the Chi-square test for qualitative data. Intra- and intergroup comparisons of pain quantified by VAS during the first 48 postoperative hours were performed by ANOVA for repeated measures.

### 2.2. Preperitoneal Continuous Infiltration

**Figure 1:**
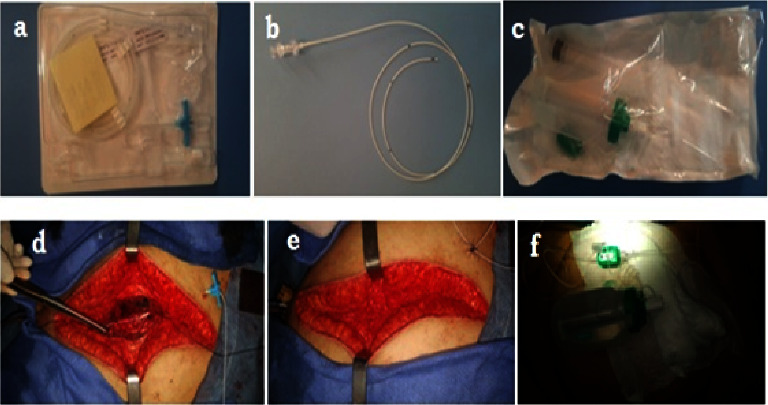
Infiltration kit and its placement in the wound after surgery (photos taken in operating block of the Hassan II University Hospital-FEZ-). (a) Catheter with its peelable introducer needle, (b) multiholed catheter allowing a homogeneous diffusion of the local anesthetic, (c) the elastomeric pump and syringe for filling, (d) multiholed catheter placed in preperitoneal position (between the parietal sheet of the peritoneum and the musculoaponeurotic layer), (e) closure of the musculoaponeurotic plane, and (f) flow adjustment and administration start of bupivacaine after wound dressing.

In the final stage of surgery and before the parietal closure, between the peritoneum and the musculoaponeurotic layer, the surgeon puts a catheter (InfiltraLong set Catheter 19G∗700 mm, multiperforated Pajunk GmbH Medizintechnology Karl-Hall-Str.1.78187, Geisingen, Germany) allowing continuous infusion of the local anesthetic (Figure 1) for the bupivacaine group and infusion of saline for the saline group. The closure of the aponeurotic and cutaneous planes did not show any particularity. Bupivacaine (solution for injection 5mg/ml, Laboratory Aguettant, France) or saline was aseptically introduced into the pump container (Fuser Pump Set for 001157-30C Pajunk GmbH Medizintechnology Karl-Hall-Str.1.78187, Geisingen, Germany); the draining of which was monitored by a tubing equipped with a flow limiter. The injection is continued constantly at a flow rate of 5 ml/hour for 48 hours, and then the catheter is removed.

### 2.3. Postoperative Analgesia and Monitoring

Good postoperative pain management after abdominal starts with an appropriate measurement of pain. In addition to continuous preperitoneal wound infiltration, multimodal analgesia included injectable paracetamol 1 g every 6 hours (Perfalgan®, 10 mg/ml B. Braun Medical SA 08191 Rubi, Espagne), Nefopam 10 mg/ml (NefopamMylan, Saint-Priest, France), and morphine parenterally if necessary. The pain was assessed by the visual analog scale (VAS). In case VAS was higher than 30/100 mm for rest pain and greater than 60/100 mm for mobilization pain, a morphine derivative was then administered (morphine sothema 10 mg/ml, Laboratoires Sothema, Bouskoura, Morocco) subcutaneously (SC) or intravenously (IV) up to three times daily.

In addition, the recovery of intestinal transit was evaluated separately by the resumption of both gaseous and solid transits. A monitoring and evaluation form, including assessment of pain by VAS and clinical signs of toxicity, was provided to the nurses. The screening for clinical signs of toxicity and the VAS at rest and mobilization were systematically carried out every six hours (when patients were asleep, they were not awakened). The VAS value retained for each postoperative day and for each patient was the average of the four values measured over the day. In addition, to evaluate the safety of the postoperative analgesia procedure, it was necessary to note any complications of the scar infiltration: premature withdrawal, catheter occlusion, infusion discomfort, or withdrawal pain.

### 2.4. Blood Sampling

The measurement of bupivacaine plasma concentration was only for the bupivacaine group. Blood samples for analyses were then obtained and taken in an EDTA (ethylenediaminetetraacetic acid) tube at definite times: T_0_ (local anesthetic injection time), T_3h_, T_6h_, T_12h_, T_24h_, T_48h_, and T_72h_. Three sampling points were used: from the elbow of the hand, from the central lane, or from the infusion route.

The plasma samples were separated by centrifugation (Centrifuge Universal 320 HettichZentrifugen) of blood samples which was analyzed immediately or stored at −20°C until use. The plasma concentration (*μ*g/ml) was measured at each instant by HPLC-DAD Shimadzu (Kyoto, Japan) at the pharmacology andtoxicology laboratory of Hassan II University Hospital, Fez, Morocco.

## 3. Results

### 3.1. Clinical and Demographic Characteristics of the Study Population

This paper evaluates a total population study comprising 60 patients (30 in the bupivacaine group and 30 in the saline group). The first part of the study concerned patients who did not receive bupivacaine infiltration (saline group), while the second part focused on all patients who received this analgesic technique (bupivacaine group). Intergroup comparison did not reveal any significant difference in age, sex, duration of intervention, type of intervention, or incision length (Table 1).

### 3.2. Data Related to Scar Infiltration and the Postoperative Period

Regarding postoperative pain management, some data are useful such as the type of tumor requiring laparotomy surgery, the duration of the procedure, the length of incisions, and the length of stay in the intensive care and surgical departments, which are shown in Tables 1 and 2. The duration of the surgical procedures for both groups varied between 3 and 8 hours, and the mean length of stay in the A4 intensive care unit and visceral surgery unit was 1.3 ± 0.7 and 8.2 ± 4.1 days for the bupivacaine group and 1.5 ± 0.8 and 8.8 ± 5 days for the saline group, respectively. For the bupivacaine group, the length of hospital stay in the postoperative intensive care unit was 24 hours for 23 patients and 48 hours for 6 patients. Only one patient was admitted to the intensive care unit for 4 days because of postoperative hypoxemia related to atelectasis.

For the saline group, the duration of hospitalization in the intensive care unit postoperatively was 24 hours for 25 patients and 48 hours for 5 patients. For both the study groups, minor hemodynamic changes and metabolic or respiratory events (apart from atelectasis) were noted during the intensive care unit stay.

For patients who had benefited from scar infiltration, no technical incident was noted during catheter placement. In the postoperative period, only two catheter withdrawals were reported: the first one due to the accidental withdrawal during positioning of the patient in the intensive care bed, and the second was justified by a bad positioning and cutting of the catheter, revealed by a permanent blocking of the perfusion of the local anesthetic. During the pain management protocol, these two withdrawals were observed in the first six postoperative hours. In the one hand, there was no reported patient pain at the abdominal catheter site during the infusion or when the infiltration catheter was removed. On the other hand, we noted no clinical or local infectious signs related to this continuous infiltration procedure. In addition, there was no evidence of clinical toxicity (hemodynamic, neurological, and respiratory) noted during the period of infusion of bupivacaine 0.25% through the infiltration catheter. Finally, there were zero deaths in both groups during the entire hospitalization period.

### 3.3. Data Related to Postoperative Pain

In clinical practice, the VAS is a commonly used and simple method for the measurement of pain intensity variations in terms of validity, reliability, and sensitivity.

**Figure 2:**
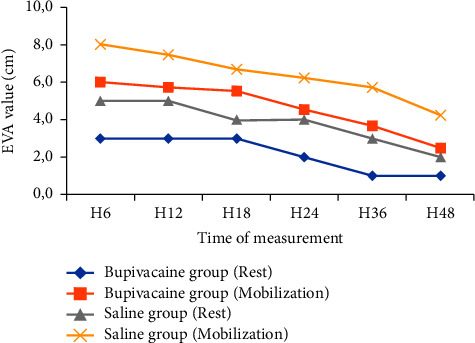
The average VAS pain scores at rest and on mobilization during the first 48 hours, assessed by the patient using a visual analog scale.

The results of daily VAS values are given in Figure 2 (at H_6_, H_12_, H_18_, H_24_, H_36_, and H_48_). If a VAS figure of less than 3/10 cm at rest and a figure of less than 6/10 cm on coughing or mobilization are considered as the threshold for satisfactory postoperative analgesia, a majority of patients were adequately relieved at rest and on mobilization for the bupivacaine group. The VAS values recorded for the saline group were significantly higher than for the bupivacaine group, reaching a maximum average at H6 of 6/10 cm at rest and 8/10 cm on mobilization. Statistically, VAS at rest or at mobilization was changed significantly over time during the first 48 postoperative hours (ANOVA for repeated measures, *F* = 22.90; *P* < 0.0001 and *F* = 29.97; *P* < 0.0001), and there was a significant difference between the two groups (*F* = 57.89; *P* < 0.0001 and *F* = 66.65; *P* < 0.0001) (Figure 2).

The mean morphinomimetic (fentanyl) consumption intraoperatively in 30 patients in each group (bupivacaine group and saline group) was 276.8 ± 48.7 *μ*g and 277.1 ± 43.8 *μ*g, respectively.

**Figure 3:**
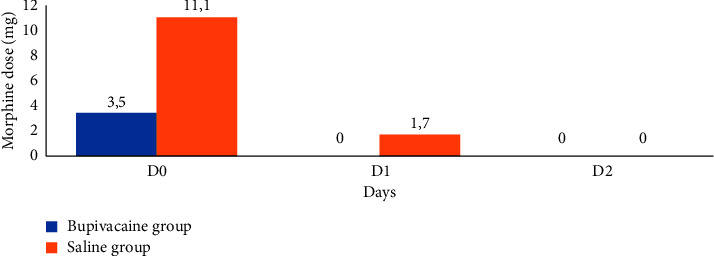
The average dose of morphine consumed for each group according to the days.

**Figure 4:**
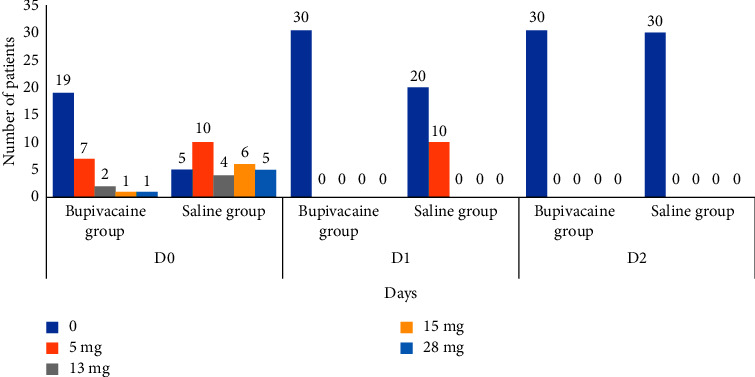
Morphine consumption during the first 48 postoperative hours.

For the bupivacaine group, morphine administration was only required during the first 24 hours (D0: day of surgery) with an average of 3.5 mg (Figure 3). Nineteen patients (63%) did not require any morphine derivative during the first 48 postoperative hours. Of the eleven patients (37%) who took parenterally morphine postoperatively, the total morphine consumption was 5 mg in seven patients, 13 mg in two patients, 15 mg in one patient, and 28 mg in one patient (Figure 4).

For the saline group, morphine administration was required on days D0 and D1 with an average of 11.1 mg and 1.7 mg, respectively (Figure 3). During D0, only five patients did not receive any morphine derivative, the total morphine consumption was 5 mg in ten patients, 13 mg in four patients, 15 mg in six patients, and 28 mg in five patients (Figure 4). During D1, twenty patients did not receive morphine while ten patients acquired 5 mg for each. During D2, no patients required morphine the day after surgery. In addition, the patients in the bupivacaine group were able to participate actively in the respiratory physiotherapy sessions with no pain-related functional limitation. A single patient presented hypoxemia on postoperative atelectasis; this was controlled by a few sessions of noninvasive ventilation, active respiratory physiotherapy, and parenterally antibiotic therapy with a positive clinical evolution. However, for the saline group, there were some side effects after administration of morphine compounds, mainly nausea and vomiting in 13 patients (43.3%) and respiratory depression in one patient (3.3%).

### 3.4. Data Related to Bupivacaine Pharmacokinetics

**Figure 5:**
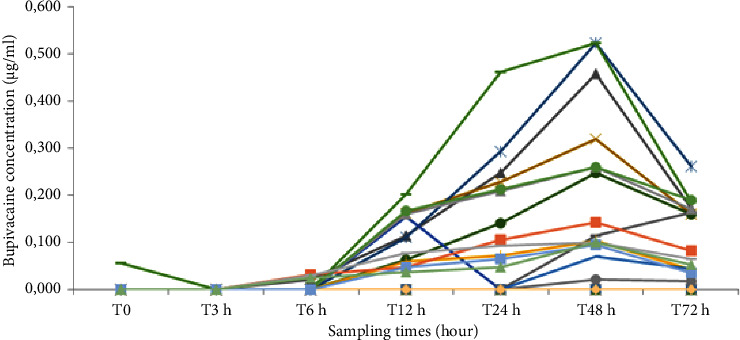
Curves of venous plasma concentrations of bupivacaine as a function of time after infiltration of the wound with 5 ml/h of bupivacaine for 48 hours in thirty patients in the bupivacaine group. Each curve shows one patient.

**Figure 6:**
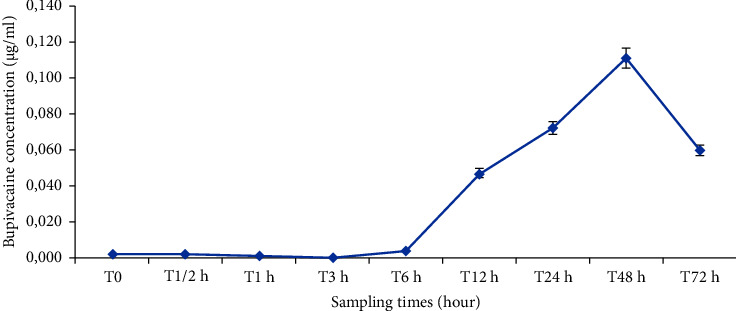
Mean plasma concentration-time profile of bupivacaine after administration by continuous scar infiltration (mean ± SD).

The dose of bupivacaine administered over 48 hours was 300 mg in the 30 patients of bupivacaine group. In most cases in this group, bupivacaine was essentially undetectable or at very low concentrations, well below the toxicity value which is around the value of 1.6 *μ*g/ml (Figure 5). We have found a pattern of increasing in plasma concentrations from the 6th hour onwards. The mean concentration was recorded at the forty-eighth hour (catheter removal time) with *C*_max_ = 0.111 ± 0.162 *μ*g/ml (Figure 6). The highest concentration (*C* = 0.522 *μ*g/ml) was reported in two patients. In most of the cases, the concentration of bupivacaine was reduced after 48 hours.

### 3.5. Data Related to Patient Comfort and Satisfaction

**Figure 7:**
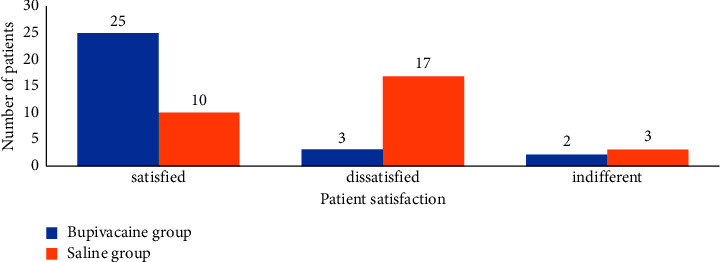
Patient satisfaction with analgesia by scar infiltration for both groups (bupivacaine group and saline group).

Concerning the patient satisfaction, among the 60 patients in our study, the recovery of gaseous and solid intestinal transit was closed for both groups. For the bupivacaine group, recovery of gaseous bowel transit was observed on day 2 in twenty-one patients, on day 3 in seven patients, and on day 4 in two patients. One patient had solid transit on day 1, ten patients on day 2, fourteen patients on day 3, two patients on day 4, and three patients on day 5. The majority of patients (83.3%) were satisfied with the analgesic strategy except for three patients. Two of these three patients experienced early withdrawal of the infiltration catheter. For the saline group, recovery of gaseous bowel transit was observed on day 2 in twenty patients, on day 3 in eight patients, and on day 4 in two patients. Eleven patients had solid transit on day 2, thirteen patients on day 3, three patients on day 4, and three patients on day 5.56.7% of the patients were not satisfied with the analgesic strategy, but 33.3% of the patients expressed satisfaction (Figure 7).

## 4. Discussion

The development of locoregional analgesia procedures is tending towards methods that do not interfere with rehabilitation and that target the operative site by eliminating the parietal component of postoperative pain. Wound infiltration with wound catheter may reply to these specifications [4]. The use of morphine has significant side effects, not only may it delay rehabilitation, but it may also promote tumor proliferation [5]. Fentanyl as a morphinomimetic has a contextual half-life of elimination, which varies between 3 and 4 hours. It was used in our study only intraoperatively for surgical procedures of the patients in our study, having as average duration of 5.7 ± 1.1 hours for the bupivacaine group and 5.5 ± 1.4 hours for the saline group. In this case, the use of fentanyl ensures analgesia until the end of the operation and covers the immediate postoperative period [14].

In our clinical study, the majority of patients in the bupivacaine group (63%) did not require morphine. The consumption of morphine in the 37% of patients was only justified on the day of surgery. On the other hand, only 17% of the patients in the saline group did not need morphine on the day of the operation. This gives an advantage to our analgesic technique used.

In the postoperative analgesia, the wound infiltration has a beneficial effect on the patient's recovery, due to analgesia and reduced morphine consumption [4]. This advantageous outcome has been demonstrated in several studies; for example, the period of hospitalization was significantly reduced by continuous injection of ropivacaine 2 mg/ml for 48 h into the sternotomy scar [14]. In another study, the use of ropivacaine 2 mg/ml by infiltration procedure for 55 h after major spinal surgery led to the same result [15]. In another study, in abdominal surgery, continuous preperitoneal infiltration of ropivacaine 2 mg/ml for 48 h led to an acceleration of the resumption of intestinal transit, a shortening of the duration of hospitalization, and an improvement in the quality of sleep during the first two postoperative nights [16]. For these benefits, scar infiltration can be considered as an analgesic technique that fits into the fast-track surgery approach [17]. Therefore, in our work, 83.3% of the patients were satisfied with this method of postoperative analgesia by infiltrating bupivacaine, 70% of the patients resumed gaseous intestinal transit on the second postoperative day, and almost all of the patients made their first rise the day after the operation. On the other hand, in the saline group, 56.7% of the patients were not satisfied with a significant use of morphine derivatives. Through our series, it is evident that the scar infiltration technique, as part of a multimodal analgesia protocol, allowed a better management of the initial postoperative period (first 48 hours) from the awakening to the stay in intensive care unit. This was evidenced by the comparison of the two groups, the quality of analgesia, the low consumption of morphine, the recovery of respiratory function, the comfort of the patients (resumption of transit and early rising), and the degree of satisfaction of the patients. All these favorable criteria eased the task of the medical team of the intensive care unit for the bupivacaine group and made it possible to transfer the patients rapidly to the surgical service to complete the management according to the neoplastic pathology.

With the presence of a saline group considered as a control group, our results showed a good efficiency of postoperative analgesia by scar infiltration of bupivacaine, with moderate pain intensity and low morphine consumption. The three main ideas to be drawn from this series of observations are the analgesic interest of bupivacaine scar infiltration through a catheter, the good tolerance of this administration pathway proved by the low serum concentrations of bupivacaine during this analgesic protocol, and the absence of local complications related to the analgesic technique. Continuous wound infiltration as postoperative analgesia is often intended for mild to moderately severe postoperative parietal surgery [13, 18]. Although in intra-abdominal surgery, the analgesic value of scar infiltration remains disputable. Despite several randomized studies which have also demonstrated the efficiency of the continuous scar infiltration method, the techniques and products used are highly variable [18, 19]. Inconsequential results have been reported [20]. They may be due to poor administration, poor choice of product, or inappropriate doses [21]. There are different data concerning the best location of the catheter in the parietal planes and the type of surgery, such as the studies carried out by numerous authors [20, 22, 23]. Furthermore, the choice of the length of the catheter is very crucial, in a way that some holes will not be found outside the scar, this is the reason for using a catheter whose diffusion zone is close to the length of the scar, which improves the homogeneous diffusion of the local anesthetic and the performance of the treatment [24]. In our study, the catheter chosen allows the entire scar to be covered, thanks to its multiperforated part measuring 22 cm, thus covering the length of all the surgical incisions made for our patients, which is on average 15.5 ± 3.1 cm for the bupivacaine group and 15.3 ± 3.9 cm for the saline group.

The infusion of local anesthetics at a low flow rate, not adapted to the size of the scar, may be the cause of technique's failure (for multiperforated catheters of 10 cm or more, the flow rate must not be less than 5 ml/h), the flow rate chosen in our study was 5 ml/h in order to not reach a toxic concentration of local anesthetic. The catheter placement has an important influence on the efficiency of the technique. Hence, in abdominal surgery, the deep catheter positioning in the front peritoneal plane can achieve the most successful results [25]. The catheter was positioned between the musculoaponeurotic plane and the peritoneum for our patients, a place deep in the scar that is more painful than the subcutaneous layer.

One study showed no interest in this preperitoneal administration [26]; it concerned gynecological surgery, where the catheter's position was not well described, and the local anesthetic was administered by repeated boluses, at a very low daily dose, which may explain the insignificant results. Thus, in our work, the choice of bupivacaine was based on its wide clinical use and its pharmacological data. Currently, the most commonly used local anesthetics are bupivacaine, ropivacaine, and levobupivacaïne [4].

However, numerous studies report severe cardiovascular toxicity induced by bupivacaine [27]. Although ropivacaine and levobupivacaine may present a more reliable alternative to bupivacaine, its use is still widespread because it provides a more lasting effect [28]. Moreover, in Morocco, bupivacaine and lidocaine remain the only products used for locoregional analgesia in adults, mainly for financial reasons. However, the very short duration of action of lidocaine limits its use, which leads to frequent use of bupivacaine, particularly in scar infiltrations. However, as with all agents in this pharmacological family, there are significant neurological and cardiac risks in the event of overdose. The dose used is lower than the highest total dose to be prescribed (400 mg in 24 h) [29].

In our prospective study, we did not find any incident of toxicity, and the blood tests carried out at D0, D1, and D2 in patients who received 300 mg of bupivacaine during the first 48 hours after surgery showed a total bupivacaine level of about 0.5 *μ*g/ml, i.e., below the upper limit of toxicity observed in patients in the case of postoperative epidural infusion [30], and also below the limit of toxicity in the operated patient. This concentration is generally considered, according to some works, to be the threshold of toxicity when it is greater than 4 *μ*g/ml [18, 31].

The therapeutic efficiency, the safety, and the side effects of surgical wound infiltration with local anesthetic for the management of postoperative pain after cesarean section have been evaluated in several studies and were generally in agreement with this analgesic technique [11, 32]. In another clinical research, the authors compared bupivacaine infiltration into the scar with epidural analgesia using repeated bolus doses of local anesthetic in both pathways [32]. They concluded that infiltration of the scar with a local anesthetic through a catheter provided analgesia comparable to epidural analgesia, clearly demonstrating the benefit, safety, and low risk of the infiltration technique.

The cost associated with the use of continuous wound infiltration for pain management after abdominal laparotomy is partially offset by a reduction in resource consumption. In terms of cost, compared to epidural analgesia, continuous wound infiltration is less costly with almost equivalent effectiveness [33]. In reviewing the literature, one study compared epidural analgesia with continuous wound infiltration with 0.2% ropivacaine, as part of the “Fast Track Surgery” approach, and concluded that epidural analgesia provided faster functional recovery than continuous wound infiltration with reduced hospital stay after open colorectal surgery [34]; however, epidural analgesia is also characterized by its technical constraints during placement and its side effects [35].

In our preliminary study, no toxic clinical effects of bupivacaine administered using continuous scar infiltration were reported. This finding is largely related to the analgesic concentration of the bupivacaine used (0.25%), the low infusion dose (5 ml/h), and the low risk of vascular passage of local anesthetics by this route of administration, which is in line with the data in the literature. Verification of the correct positioning of the catheter by the surgeon in the operating wound is another guarantee of the effectiveness and the safety of the technique. Consequently, adverse events related to continuous wound infiltration techniques are limited or absent. The absence of a motor block allows for rapid mobilization of patients, optimizing the postoperative rehabilitation process, and reducing the rate of complications related to perioperative management in a neoplastic patient population. These techniques do not require special monitoring and patients can be safely reintegrated into the conventional hospital setting immediately after surgery, which is a major advantage. There are no reports of wound healing problems, and the incidence of surgical site infection does not appear to be increased by the use of multiperforated catheters. The use of large volume elastomeric pumps should be recommended, as it reduces the number of operations required to fill the infusion syringes, and thus the risk of septic inoculation.

## 5. Conclusion

Overall, this study strengthens the idea that continuous wound infiltration is a simple, safe, and well-tolerated analgesic technique that can be offered to all patients. It improves pain control at rest and during mobilization and reduces the consumption of morphine derivatives and their associated side effects. It accelerates recovery after laparotomy digestive surgery. At the recommended dose, the risk of local infection is not available, and the combined risk of toxicity associated with the use of bupivacaine is limited.

## Figures and Tables

**Table 1:** Demographic and clinical characteristics of patients (*n* = 60).

	Bupivacaine group (*n* = 30)	Saline group (*n* = 30)	*P* value
Residential location			0.25
Urban	25	27	
Rural	5	3	
Sex			0.53
Men	14	13	
Women	16	17	
Age	55 ± 14	53 ± 13	0.47
ASA score			0.16
ASA_I_/ASA_II_/ASA_III_/ASA_IV_	18/8/3/1	22/4/4/0	
Diagnostic			0.47
Ampullome vaterian	7	6	
Tumor of the pancreatic head	5	6	
Colon tumor	4	3	
Gastric tumor	4	4	
Esophagus tumor	1	0	
Duodenal tumor	1	2	
Sigmoidal and rectal tumor	7	7	
Gall bladder tumor	1	2	
Duration of intervention (h)	5.7 ± 1.1	5.5 ± 1.4	0.51
Incision length (cm)	15.5 ± 3.1	15.3 ± 3.9	0.88

ASA: American Society of Anesthesiologists (physical status classification system).

**Table 2:** The average length of stay in the intensive care and visceral surgery units for both study groups (mean ± SD).

	Bupivacaine group (*n* = 30)	Saline group (*n* = 30)	*P* value
Hospitalization in the intensive care unit (days)	1.3 ± 0.7	1.5 ± 0.8	0.85
Hospitalization in visceral surgery unit (days)	8.2 ± 4.1	8.8 ± 5	0.62

## Data Availability

The data used to support the findings of this study are available from the corresponding author upon request.
